# Decision-Making in Geriatric Patients with End-Stage Renal Disease: Thinking Beyond Nephrology

**DOI:** 10.3390/jcm8010005

**Published:** 2018-12-20

**Authors:** Faheemuddin Azher Ahmed, Angela Georgia Catic

**Affiliations:** 1 OSF Saint Anthony Medical Center, 5666 E State St, Rockford, IL 61108, USA; 2 Baylor College of Medicine, One Baylor Plaza, Houston, TX 77030, USA; Angela.Catic@va.gov; 3 Michael E. DeBakey VA Medical Center, 2002 Holcombe Blvd, Houston, TX 77030, USA

**Keywords:** dialysis, palliative, older adults, transplantation

## Abstract

Compared to younger individuals, the prevalence of end-stage renal disease (ESRD) in elders is notably higher. While renal replacement therapy, usually with hemodialysis, is accepted therapy in younger patients with ESRD, decisions regarding the treatment of advanced kidney disease in the elderly population are more complex, secondary to the physiologic changes of aging, concurrent geriatric syndromes, and varying goals of care. Evaluation for possible initiation of dialysis in geriatric patients should be multidisciplinary in nature and patient-focused, including a consideration of physical, cognitive, and social function. If renal replacement therapy is not pursued, optimization of medical management or symptom management needs to be the goal of care.

## 1. Introduction

Compared to younger individuals, the prevalence of end-stage renal disease (ESRD) has increased more significantly among elders over the last several years. At the present time, the prevalence per million of ESRD is highest among individuals aged 65–74 years and the incidence rate is the highest among those 75 years of age and older [1]. The discrepancy among younger adults and the geriatric population is secondary to longer survival among the ESRD population, resulting in the aging of affected patients, as well as increasing numbers of individuals aging with chronic conditions that lead to ESRD (i.e., type 2 diabetes mellitus and hypertension). Given the increasing population of geriatric patients with ESRD, increased consideration must be given to appropriate treatment of the condition in this population. Primary care providers are instrumental in guiding patients and their families in the thoughtful consideration of an individualized, patient-centered approach to ESRD treatment. Treatment considerations for ESRD should take into account the risks/benefits of renal replacement options; the impact of the physiologic changes of aging, medical comorbidities, and geriatric syndromes, including frailty; and options for kidney supportive care.

### 1.1. Renal Replacement in the Elderly

Renal replacement therapy is commonly pursued in patients with ESRD. Among patients with ESRD in the United States, 63.2% are treated with hemodialysis, 7.0% with peritoneal dialysis, and 29.6% with a kidney transplant [1]. Data registries from around the world indicate that many of the patients being initiated on dialysis are elderly. According to the UK Renal Registry (UKRR), the number of incident dialysis patients (both hemodialysis and peritoneal dialysis) was highest among individuals 65 to 74 years of age [2]. In Canada, Europe, and Australia/New Zealand, a significant percentage of patients initiating dialysis are 65 years of age or older (84%, 56%, and 46%, respectively) [3,4,5]. In the United States, 49% of patients initiating renal replacement therapy are 65 years of age or older [1]. Among this cohort, the vast majority pursue hemodialysis (90.0% patients 65–74 years and 94.0% patients 75+), while lower numbers are treated with peritoneal dialysis (8.4% patients 65–74 years and 5.9% patients 75+) or renal transplant (1.6% patients 65–74 years and 0.1% patients 75+). 

In the young adult population, the benefits of dialysis include an increased life span, symptom relief, and an improved quality of life. Dialysis-associated outcomes are more variable in older adults but, in general, geriatric patients are at an increased risk for poor outcomes. In addition, elders undergoing dialysis have higher mortality rates compared to younger individuals receiving renal replacement therapy. Among men receiving dialysis, deaths per 1000 patient-years were 144 for those ages 45–64 years, 249 for those 65–74 years, and 382 for those 75+ [1]. A similar increase in mortality is observed among female dialysis patients as they age. Elders ≥75 years with ESRD also have significantly higher mortality rates than members of the general Medicare population with other chronic conditions but no ESRD (i.e., 1.7 times higher than those with heart failure, 4.0 times higher than those with diabetes mellitus) [1]. Factors which impact morbidity and mortality in elders with ESRD on dialysis include the physiological changes of aging; comorbid conditions; and the presence of geriatric syndromes, especially frailty. Given the complexity of geriatric nephrology patients, the management of ESRD should entail a collaborative approach between the primary care provider, nephrologists, and geriatricians [6]. A multidisciplinary approach is needed in dialysis decision-making in geriatric ESRD population as outlined in Table 1.

### 1.2. Renal Replacement Considerations 

When considering possible renal replacement therapy in geriatric patients, providers should consider the pros/cons of each treatment modality and the impact these may have on the elder’s overall health. Consulting with a nephrologist and geriatrician can be valuable in clarifying these issues. Considerations with hemodialysis include the choice of vascular access, resources associated with the therapy, and associated complications. When considering vascular access options, clinicians should take into account the life expectancy of the patient. While arteriovenous fistulas (AVFs) are generally preferred to arteriovenous grafts (AVGs) given the lower infection risk and relative ease of maintaining patency, they take longer to mature and there is an increased risk of non-maturation with increasing age [7,8]. AVGs may be a more reasonable access option in frail elders with limited life expectancy, although should be reviewed based on an individual patient’s co-morbidities because of their higher infection rates and an increase in the cardiac overload. Resources required for hemodialysis should also assessed when considering this treatment in elders. Many geriatric patients and their families find the regular travel and significant time commitment associated with hemodialysis to be overly burdensome. Finally, elders receiving hemodialysis are at an increased risk of complications, including hemodynamic instability, depression, cognitive decline, malnutrition, and infections [9]. 

Clinical practice guidelines recommend against prescribing less than thrice-weekly dialysis for patients without substantial residual renal function [10]. There have been no randomized controlled trials that studied dialysis dose in patients with substantial residual renal function. Residual renal function has been shown to improve overall health and well-being and its decline has been shown to be a strong predictor of mortality [11]. The concept of incremental dialysis has been emerging, which offers opportunity to expand dialysis frequency and individualize dialysis therapy. Among incident HD patients with substantial residual renal function, incremental dialysis may be a better option to preserve renal function and a better health-related quality of life, which are among the strongest predictors of survival in the first year of dialysis [12]. In older adults, an incremental dialysis approach starting with one to two dialysis sessions per week can help them adapt to the dialysis. Dialysis prescription can vary in each individual patient based on multiple co-morbidities, tolerance, and other factors. A personalized approach to dialysis prescription, as proposed by Piccoli et al., can help achieve a more patient-centered approach in older adults initiating dialysis [13].

When considering peritoneal dialysis in elders, barriers to participation and relative contraindicates must be considered. Compared to hemodialysis, peritoneal dialysis requires significant technical participation from the patient or caregiver. Elders with significant impairment of cognition, vision, or dexterity would be poor candidates for peritoneal dialysis unless a caregiver could provide significant assistance with the process. However, if elders are able to participate in peritoneal dialysis independently or with assistance, they may prefer this method as it does not require them to leave their home. Medical conditions which are a relative contraindication to peritoneal dialysis include severe pulmonary disease; significant scarring from previous abdominal surgery; uncorrectable hernias; active inflammatory bowel disease; and colostomy, ileostomy, or gastric tubes [14]. 

The results of studies comparing mortality between hemodialysis and peritoneal dialysis in older adults have been mixed [15,16]. Data regarding short-term mortality is limited in the United States due to a lack of Medicare reporting as they do not become the principal payer until 90 days after dialysis initiation. Studies have found increased mortality among diabetic patients receiving peritoneal dialysis compared to hemodialysis [17]. A meta-analysis done in Korean patients by Han et al. showed a higher death rate in elderly PD patients than those on HD [18]. There was no difference in the quality of life and physical function between PD and HD patients in the age group of 60 plus [19].

Kidney transplantation, while performed less frequently than dialysis in the geriatric population, has been shown to increase life expectancy and improve quality of life compared to dialysis [20]. In a study by Wolfe et al. comparing mortality in wait-listed dialysis patients versus recipients of a first cadaveric transplant, the transplant recipients demonstrated improved longevity at all ages, including patients who were 60 to 74 years of age [21]. Another study showed a 41% overall adjusted relative risk of death for transplant patients in comparison to wait-listed dialysis patients [22]. Determining who would derive the greatest benefit from transplants given the shortage of donor organs will continue to be an important ethical consideration as increasing numbers of individuals survive into advanced old age with ESRD. 

## 2. Physiologic Changes of Aging

Physiological changes normally occur with the aging process and involve the majority of organ systems in the body. Anatomic and physiologic changes occur in the kidneys, leading to a decline in renal function of 0.75 mL/min per year, even in the absence of relevant comorbidities (i.e., hypertension and diabetes) [23]. Anatomic changes include a decrease in renal mass; decreased glomeruli and glomerular structural changes; tubulointerstitial fibrosis, scarring, and infarction; and changes in vascular responsiveness and autoregulation [24,25]. Common physiologic changes include a decreased blood flow, glomerular filtration rate (GFR), and diluting and concentrating capacity [24].

In addition to physiologic changes of aging, which can contribute to ESRD, changes in the other organ systems can significantly impact the ability of elders to tolerate dialysis. Cardiovascular changes with aging can result in significant hemodynamic alterations during dialysis and lead to either intolerance or inefficient dialysis. This can influence mortality, morbidity, and functional status.

In a study done by Luca De Nicola to define if age modifies the prognosis of CKD patients on nephrology care, the rate was double for the end point of ESRD and death without ESRD in patients over the age of 75 years [26].

### 2.1. Medical Co-Morbidities

Many older adults suffer from multiple chronic conditions, with 68.4% of Medicare beneficiaries having ≥2 and 36.4% having ≥4 [27]. In addition to ESRD, common chronic diseases in the geriatric population include chronic pulmonary disease, coronary artery disease, and type 2 diabetes mellitus. The presence of comorbid chronic disease can significantly impact dialysis outcomes. There are several decision tools which can assist providers in predicting prognosis and thus inform decisions regarding the treatment of ESRD.

1. nCI (new comorbidity index): This comorbidity score assigns a numerical weight to the following comorbid conditions: (1) One point each-Diabetes mellitus and coronary artery disease; (2) Two points each-Cerebral vascular accident, peripheral vascular disease, other cardiac disease, dysrhythmia, chronic obstructive pulmonary disease, GI bleeding, liver disease, and cancer; and (3) Three points to congestive heart failure. The patient’s total comorbidity score is calculated by summing the points for each comorbidity present. The index was successful in analyses for mortality, hospitalization, and medical costs [28]. Age was not included in the nCI. However, Kan et al. implemented the index in patients 65 years or older with ESRD on hemodialysis or peritoneal dialysis and determined that the nCI was a strong predictor of mortality, even without a consideration of age [29].
2. The modified Charlson (MCS) score, adapted from the original Charlson Comorbidity Index, is useful in identifying ESRD patients with a poor prognosis. The Charlson Comorbidity Index was initially derived in medical inpatients and included 19 comorbid conditions assigned the following weights: (1) 1 point-Myocardial infarction, congestive heart failure, peripheral vascular disease, cerebral vascular disease, dementia, chronic lung disease, rheumatological, peptic ulcer disease, mild liver disease, and diabetes without complications; (2) 2 points-Hemiplegia, diabetes with complications, neoplasia, leukemia, lymphoma, and renal disease; (3) 3 points-Moderate/severe liver disease; and (4) 6 points-Metastatic disease and human immunodeficiency virus [30]. It has been translated to multiple patient populations, including being used to predict mortality, inpatient costs, and length of hospital stay [31,32]. After analysis, the following comorbidities were included at adjusted weights: (1) 1 point-Peripheral vascular disease, dementia, chronic lung disease, rheumatological, peptic ulcer disease, and diabetes with complications; (2) 2 points-Myocardial infarction, congestive heart failure, cerebral vascular disease, diabetes without complications, moderate/severe liver disease, and leukemia; (3) 5 points-Lymphoma; and (4) 10 points-Metastatic disease. The modified Charlson score was more effective than the original index in predicting survival in patients with ESRD.
3. Couchoud et al. developed and validated a prognostic score in ESRD patients ≥75 years who were initiating dialysis to predict six-month mortality [33]. Risk factors included in the score were body mass index, diabetes, congestive heart failure, peripheral vascular, disease, dysrhythmia, active malignancy, severe behavioral disorder, total dependence for transfers, and initial context. Mortality rates increased with higher numbers of points on the risk scale, ranging from 8% in individuals with 0 points to 70% in those with 9 points.
4. Thamer et al. developed a dialysis initiation risk score for older adults (≥67 years) with ESRD [34]. The tool uses commonly available information (age, low/unknown albumin, need for assistance in daily living, living in nursing home, cancer, heart failure, and prolonged/frequent hospitalization) to predict three- and six-month mortality. Patients with a median score using this tool had a 12% mortality risk at three months and a 20% risk at six months. Among those with the highest scores ≥8), the mortality risk increased to 39% at three months and 55% at six months.

### 2.2. Geriatric Syndromes

Geriatric syndromes, often defined as a multifactorial condition involving the interaction between identifiable stressors and age-related factors, often involve multiple organ systems and can lead to significant disability [35,36]. Common geriatric syndromes include cognitive impairment, urinary incontinence, falls, and frailty. Given that geriatric syndromes generally have a significant impact on the quality of life of the elder, as well as their caregivers, a consideration of their goals and the development of an individualized management plan is paramount in the management of geriatric syndromes and concurrent renal dysfunction. 

Although cognitive impairment is common in older adult patients with chronic kidney disease, it is often poorly recognized and under diagnosed. Studies have demonstrated that hemodialysis can lead to increasing cognitive dysfunction, involving difficulty with decision-making and self-care, including medical self-management (i.e., following medication and dietary instructions) [37,38,39]. Screening elders with ESRD for cognitive issues, using a standardized evaluation such as the Montreal Cognitive Assessment (MoCA), can help inform treatment decisions, including increasing support at home and referring to geriatric medicine if warranted. 

Unintentional falls are the leading causes of nonfatal injuries among elders ≥65 years, with >3 million occurring in 2016 according to the National Center for Injury Prevention and Control, CDC. During that year, falls resulted in 29,668 deaths in geriatric individuals. Falls in older adults are most often multifactorial in nature, with risk factors including visual deficits, orthostasis, gait instability, and medication side effects. Among dialysis patients, the risk for falls and fall-related consequences is higher, secondary to additional predisposing factors, including dialysis-related hemodynamic, clinical, and functional changes; increased rates of polypharmacy; and a high prevalence of comorbidities, including diabetes, cardiovascular disease, and neuropathy. In a longitudinal study of fall risk among dialysis patients, 47% of patients had a fall over a median of 468 days, with a fall rate of 1.60 falls/person-year of follow-up and an average of 2.78 falls per person [40]. In addition, the incidence of hip fracture among dialysis patients is four times higher than age-matched cohorts. Therefore, the increased incidence of falls and fall-related injuries in dialysis patients is an important risk factor for poor quality of life and dependency. 

A comprehensive geriatric assessment can be very helpful in identifying geriatric syndromes, considering the implication these will have on ESRD and possible treatment options, and predicting and planning for issues which may arise in the future. 

### 2.3. Frailty

Frailty, a common issue in older adults with ESRD, is commonly defined as a physiologic state of increased vulnerability to stressors due to a decreased physiologic reserve [41]. The most commonly used models of frailty are the phenotypic model and the cumulative deficit model. The phenotypic model, developed by Fried and colleagues, used data from participants ≥65 years who participated in the Cardiovascular Health Study [42]. Frailty was defined as a clinical syndrome where three or more of the following findings were present: unintentional weight loss, self-reported exhaustion, weak grip strength, slow gait speed, or low physical activity. The frailty phenotype was more common with increasing age and independently predicted incident falls, worsening physical function, hospitalization, and death. The cumulative deficit model is based on the Frailty Index (FI), which was developed by Rockwood and colleagues using data from the Canadian Study of Health and Aging (CSHA) [43]. Ninety-two baseline symptoms, signs, laboratory values, disease states, and disabilities were evaluated in each subject and their frailty index was calculated based on the presence or absence of these variables, all of which have equal mathematical weight. Unlike the frailty phenotype, the cumulative deficit model presents a more graded model of frailty as patients experience a progressive accumulation of deficits. Subsequent studies have successfully calculated the FI with fewer variables (30–40) and through patient self-reporting without a loss of predictive validity [44,45].

Studies evaluating frailty in individuals with chronic kidney disease have determined that the prevalence increases as the glomerular filtration rate (GFR) declines and, when frailty was determined by poor self-reported physical functioning, exhaustion/fatigue, low physical activity, and undernutrition, 2/3 of incident dialysis patients qualified as frail [46,47]. The presence of frailty in incident dialysis patients is associated with an increased risk of death (HR 2.24) and of death or hospitalization (HR 1.90) [47]. While a trend toward initiating dialysis earlier in the course of ESRD has been noted over the past 20 years, including among frail individuals, there is no data to suggest that frailty improves when dialysis is instituted [48,49]. These findings suggest that it is important for clinicians to evaluate elders with chronic kidney disease for frailty and support them in making an informed decision about which treatments they would wish to pursue if their condition progresses to ESRD. 

## 3. Conservative Management/Patient-Centered Palliative Care

While there is generally a survival benefit for dialysis patients when compared to patients with ESRD who receive conservative management, this is often at the expense of patient goals, values, and lifestyle. Patients on dialysis spend a significant amount of time interacting with the medical system (i.e., dialysis, hospitalizations), which can result in physical and psychosocial stressors leading to a lower quality of life and increased risk of dying in the hospital. In a comparison of life expectancy in patients with ESRD, Carson et al. found a median survival of 37.8 months for renal replacement compared to 13.9 months with maximum conservative management [50].

A patient-centered, palliative care approach should be offered to patients with ESRD, with the goal of providing care which aligns with the patient’s goals and values. This may involve palliative dialysis and/or symptomatic treatment of chronic kidney disease symptoms. Palliative dialysis is aimed at improving quality of life by treating symptoms and distress. It may involve modification of duration and/or frequency of dialysis sessions [51]. 

Symptom management is aimed at ameliorating symptoms associated with chronic kidney disease. These include pruritus, sleep disorders, restless legs, dyspnea, anxiety, agitation, depression, nausea, muscle cramps, and pain.

There may be scenarios which are confounded by prognostic uncertainties and a lack of clear goals from the patient and/or family. In these scenarios, a time-limited trial of dialysis may be indicated. A time-limited trial of dialysis is defined as a goal-directed trial of renal replacement therapy with set outcomes assessment at predetermined intervals [52]. This will help patients, families/caregivers, and providers to evaluate the response to renal replacement therapy and decide on future treatments which best support the goals of care.

## 4. Bioethicist Perspective

### 4.1. Patient

Patients base their decisions on multiple factors, including personal values, beliefs, disease perception, social support, interpersonal relationships, autonomy, expected suffering, informed prognosis, and outcomes [53]. Although disease course and complications in chronic kidney disease differ from cancer and other terminal illnesses, patient preferences and end-of-life discussions should be carried out in a similar manner, with a special focus on the disease/symptom trajectory.

A systematic review by Murray et al. to identify factors influencing patient participation in the decision-making process determined that there was less focus on patient decision-making needs. In a study by Davison, around 90% of the patients reported that their nephrologist did not discuss the prognosis with them. It also reported that in around 60% of the cases, the choice to pursue dialysis was either their physician or family’s wish. The study also found that the majority of dialysis patients regretted their dialysis decision. Morton et al. concluded that the factors which make a patient choose dialysis over conservative care were an increase in life expectancy, dialysis treatment in the day or evening, and transportation ease. 

A patient-centered treatment plan should be outlined and aligned with the patient’s goals and values in conjunction with prognostication information. Holly and Schell’s review article provides a decision-making guide for nephrologists, including different questions to address while exploring goals for patients. It also guides in preparing patients and families at different stages of the disease process. 

### 4.2. Caregiver/Family

Older adults with medical comorbidities are often limited in their activities of daily living, as well instrumental activities of daily living. The same is true for End-Stage Renal Disease patients, who may require even more assistance once dialysis is added to their treatment plan. The burden and adverse effects on the family and/or caregivers is not just limited to physical impacts, but also may affect emotional, psychosocial, financial, and quality of life arenas.

In a study by Belasco et al. to assess quality of life in caregivers of older adult patients on dialysis, a significant burden and adverse effects on quality of life were experienced, especially in those on peritoneal dialysis therapy [54]. It was found that thirty-two percent showed signs of depression. A cross-sectional multicentric study done in Spain found that the caregivers of older adult dialysis patients had a worse health-related quality of life and higher risk of depression [55]. These effects were even worse if there was low social support.

Health care providers should identify potential caregivers and formulate a plan which minimizes added caregiving burden. In addition, caregivers should be educated about the different aspects/activities involved in dialysis. They can also be trained in structured coping skills to help reduce the stress and burden of caregiving. A social support system should be established. Caregivers should also be monitored periodically for any impact in their physical, functional, and mental quality of life. 

### 4.3. Advance Care Planning

Clinical practice guidelines have recommended advance care planning (ACP) in dialysis patients [56]. Nephrologists should lead the discussions in ACP. The documents that are involved in advance care planning are known as advance directives and include Living Will (LW), Durable Power of Attorney for Health Care (DPAHC), and Physician Orders Life Sustaining Treatment (POLST). This can help the decision-making process at the end of life in ESRD patients, especially withdrawal from dialysis.

## 5. Conclusions

The number of older adults is rapidly increasing across the world. It is projected that 98 million adults will be 65 years of age or older by 2060 [57]. With aging, there is an increase in overall health care expenditure and, in general, chronic kidney disease is one of the most fiscally burdensome conditions. In 2013, the cost for chronic kidney disease care among Medicare beneficiaries 65 years of age and older exceeded 50 billion [58]. 

Dialysis increases life span, provides symptom relief from fluid overload and toxins, improves quality of life, and can help fulfill personal wishes. Dialysis also comes with side effects and complications, especially in patients with multiple co-morbidities. Age by itself is not a contra-indication for dialysis. However, when coupled with multiple co-morbidities, geriatric syndromes, and functional impairment, it may not be the best treatment decision for all patients. In fact, among this population, dialysis can result in a reduction in quality of life. Therefore, the decision-making process regarding dialysis in the geriatric population should be multi-disciplinary in nature and focus on individual patient/family goals. 

## Figures and Tables

**Table 1:** Multidisciplinary Approach to Geriatric ESRD Patients.

**Nephrology Aspects**
Renal Replacement Therapies: Dialysis: Hemodialysis, Peritoneal Dialysis; Incremental Dialysis, Kidney Transplantation
Hemodialysis (HD) versus Peritoneal Dialysis (PD): Mixed results regarding mortality between HD and PD
Kidney Transplantation: Organ allocation dilemma/ethical considerations
**Geriatric/Internal Medicine Aspects**
Medical Co-morbities (Different tools and scoring systems for prognostication): Modified Charlson Score, Cohen Prognostic Model, Couchoud et al. clinical score, DOPPS, VES-13
Geriatric Syndromes: Cognitive impairment, Falls, Dizziness, Incontinence, Depression, Frailty
Impact of Dialysis on Geriatric Syndromes: Worsening of symptomatology, falls, cognitive function, frailty, depression
Physiological Changes with Aging: Functional decline in kidney function, Organ systems ageing (cardiovascular, neurologic) with more hemodynamic alterations
**Bioethicist Aspects**
Patient factors: Personal values/beliefs, Disease perception, Social support/interpersonal relationships, Autonomy, Expected suffering/difficulties and death, Informed prognosis and outcomes
Caregiver/family factors: Burden consideration—emotional, psychosocial, financial, quality of life; Support system
**Palliative Care Aspects**
Conservative Management: Symptom management, Time spent at home Incremental Dialysis
Time Limited Trial of Dialysis: Set outcomes at intervals
